# The Effect of Knee Extension Limitation on Lumbar Intervertebral Disc Compression Force During Walking: A 3D Musculoskeletal Analysis

**DOI:** 10.3390/s25216605

**Published:** 2025-10-27

**Authors:** Yuhei Kotaki, Daisuke Kudo, Ryota Kimura, Yuji Kasukawa, Hiroaki Kijima, Hidetomo Saito, Michio Hongo, Takehiro Iwami, Naohisa Miyakoshi

**Affiliations:** 1 Department of Orthopedic Surgery, Akita University Graduate School of Medicine, 1-1-1 Hondo, Akita 010-8543, Japan; ykotaki@med.akita-u.ac.jp (Y.K.); rkimura@med.akita-u.ac.jp (R.K.); hkijima@med.akita-u.ac.jp (H.K.);; 2 Department of Rehabilitation Medicine, Akita University Hospital, 1-1-1 Hondo, Akita 010-8543, Japan; 3 Department of Physical Therapy, Akita University Graduate School of Health Sciences, 1-1-1 Hondo, Akita 010-8543, Japan; 4 Department of Systems Design Engineering, Faculty of Engineering Science, Akita University Graduate School of Engineering Science, 1-1 Tegata Gakuen-cho, Akita 010-8502, Japan

**Keywords:** knee–spine syndrome, gait, peak compression force, sagittal alignment, AnyBody, musculoskeletal modeling, older adults, lumbar spine

## Abstract

**Highlights:**

**What are the main findings?**

- Peak compression force at the upper lumbar intervertebral discs during walking is significantly increased in patients with knee extension limitations.
- Mean compression forces across the gait cycle remain unchanged in patients with knee extension limitations.

**What are the implications of the main findings?**

- The findings underscore the heightened risk of disc overload during everyday activities in patients.
- Clinically assessing and managing knee extension can potentially mitigate adverse upper lumbar loading and reduce the risk of degenerative progression.

**Abstract:**

Knee–spine interactions suggest that limited knee extension may elevate spinal loading during ambulation in older adults. This study aimed to estimate lumbar intervertebral disc loads during walking in patients with knee extension limitations using a 3D musculoskeletal model and examine their relationship with sagittal alignment. Seventeen adults with radiographic knee osteoarthritis (Kellgren–Lawrence ≥ 2) underwent whole-spine lateral radiography and 3D gait analysis with force plates. A patient-scaled AnyBody model was used to compute intervertebral disc compression forces (T12/L1–L5/S1). Participants were categorized into two groups based on knee extension angle (KEA): a limitation cohort (deficit ≥ 10°) and a non-limitation cohort (<10°). Peak compression force (PCF) and mean compression force were normalized to body weight. The limitation group showed a smaller pelvic incidence and a larger sagittal vertical axis. PCF was significantly increased at the thoracolumbar and upper lumbar levels (T12/L1, L1/2, L2/3, and L3/4), whereas the mean forces remained unchanged. Knee extension limitation is associated with higher peak upper lumbar disc loading during gait, supporting the targeted management of knee extension in older adults at risk of spinal degeneration.

## 1. Introduction

Adult spinal deformity (ASD) has emerged as a critical issue in the context of an aging population, as both the incidence and severity of degenerative spinal conditions continue to rise [1]. In community-dwelling older adults, reduced extensor strength in both the back and knees has been correlated with compensatory alterations in sagittal alignment, indicating the central role of neuromuscular decline in postural adaptation [2]. This decline often presents as hyperkyphosis, a deformity associated with reduced quality of life, decreased functional independence, and a higher risk of disability in older persons [3]. Longitudinal observations indicate that once sagittal malalignment develops, deformity may progress [4]. Quality-of-life reduction and disability have been documented in middle-aged and elderly people with postural deformities and limited spinal mobility [5], and lumbar spondylosis with weak trunk muscle strength is associated with an increased fall risk [6]. Taken together, these factors help explain why corrective surgery is often pursued in ASD despite well-documented complication rates, including neurological deficits [7].

Sagittal malalignment not only compromises spinal balance but also affects the pelvis, hips, and knees, underscoring the integrated nature of the musculoskeletal system. Whole-body analyses have demonstrated that deviations in the sagittal alignment relative to the gravity line disrupt the coordinated balance of the axial skeleton [8]. Therefore, the evaluation of sagittal balance must consider the entire kinetic chain, from the spine to the lower extremities [9]. Spinal kyphosis, weaker back and/or grip strength, and slower 10 m gait time were associated with worse postural balance and a higher one-year fall rate [10]. Modeling studies and in vivo measurements have consistently indicated that such malalignment elevates paraspinal muscle demands, alters hip joint contact forces, and disrupts normal gait kinematics [11]. These biomechanical cascades illustrate the manner in which local deformities propagate system-wide effects.

Within this broader context, the concept of “knee–spine syndrome” has gained traction as a framework linking knee extension limitation to lumbar malalignment. Clinical studies have shown that limited knee extension is strongly associated with diminished lumbar lordosis [12] and that chronic knee flexion contractures contribute to spinal imbalance under both static and dynamic conditions [13]. Experimental gait analyses further support this relationship, demonstrating that artificially induced knee extension limitation leads to an exaggerated forward trunk inclination during walking [13]. More recent investigations into unilateral knee extension limitations confirm that lumbar loading metrics significantly increase during gait, further reinforcing the biomechanical link between the knee and spine [14]. In contrast to prior simulation-based studies in healthy participants, the present study enrolls patients with radiographically confirmed osteoarthritis and passive knee-extension deficit. It quantifies walking-related intervertebral disc compression from T12/L1 to L5/S1 and examines associations with spinopelvic morphology. In this study, ‘spinal imbalance’ refers to sagittal malalignment with greater positive sagittal vertical axis, pelvic retroversion (increased pelvic tilt with reduced sacral slope), and a mismatch between pelvic incidence and lumbar lordosis (PI–LL).

Most earlier studies evaluated these mechanisms in static standing postures, whereas accumulating evidence highlights that vertebral loading during walking often exceeds that observed during quiet standing. Data from telemeterized implants have revealed that spinal loads increase sharply during ambulation, producing transient peaks that are not observed under static conditions [15]. These findings highlight the need to examine spinal loading under dynamic conditions that reflect daily life. Forward trunk inclination increases proximal extensor moments via segmental moment arms, and this effect may be accentuated in low–pelvic incidence morphotypes. Standing models further relate greater SVA to elevated upper lumbar loading, motivating our focus on upper levels.

Musculoskeletal modeling is a noninvasive method for quantifying spinal forces during motion. The AnyBody Modeling System allows the integration of patient-specific kinematics, ground reaction forces, and optimization-based algorithms to estimate internal loads [16]. Given that direct in vivo measurement of intervertebral disc compression is rarely feasible, this modeling framework enables a deeper understanding of spinal biomechanics during gait. Based on these insights, we hypothesized that knee extension limitation would specifically increase the lumbar intervertebral disc compression forces during walking, with the greatest influence expected at the upper lumbar levels. The study, therefore, aimed to estimate lumbar intervertebral disc loads during walking in patients with knee extension limitations using a 3D musculoskeletal model and to examine their relationship with sagittal alignment.

## 2. Materials and Methods

### 2.1. Study Design and Ethics

This cross-sectional study adheres to the STROBE statement; the completed checklist is provided as Supplementary File S1. This study was conducted at a single academic medical center. The study protocol was reviewed and approved by the institutional ethics committee in accordance with the Declaration of Helsinki (approval code 2946; date of approval: 24 February 2023). Written informed consent was obtained from all participants before enrollment.

### 2.2. Participants and Eligibility

Seventeen ambulatory adults with radiographic knee osteoarthritis were recruited from the outpatient clinic. Inclusion criteria were: age ≥ 50 years; knee osteoarthritis graded as Kellgren–Lawrence grade ≥ 2 on standard radiographs [17]; and ability to walk independently on level ground. The exclusion criteria were prior knee arthroplasty, history of spinal surgery, presence of two or more vertebral fractures, neurological disorders affecting gait, or pain severe enough to preclude independent ambulation. Demographic data (age, sex, height, and weight) and passive knee extension angle (KEA) of both limbs were recorded by a single orthopedic specialist using a handheld goniometer. Negative KEA values denoted an extension deficit (i.e., flexion contracture). Standing whole-spine lateral radiographs were obtained to compute sagittal spinopelvic parameters.

### 2.3. Radiographic Measurements

From the full-length lateral radiographs, the following sagittal spinopelvic parameters were measured using standard definitions: sagittal vertical axis (SVA), lumbar lordosis (LL), pelvic tilt (PT), pelvic incidence (PI), sacral slope (SS), and PI–LL mismatch [18]. The measurement accuracies for these spinopelvic parameters in whole-spine lateral standing radiographs have been reported and are considered in protocol designs [19]. Spinopelvic morphology was categorized into Roussouly Types 1–4 based on the sacral slope and location of the lumbar lordosis apex [20]. All measurements were performed by the same trained assessor using a digital measurement workstation, and repeat readings were cross-checked by a second rater for gross inconsistencies

### 2.4. Gait Acquisition and Pre-Processing

**Figure 1:**
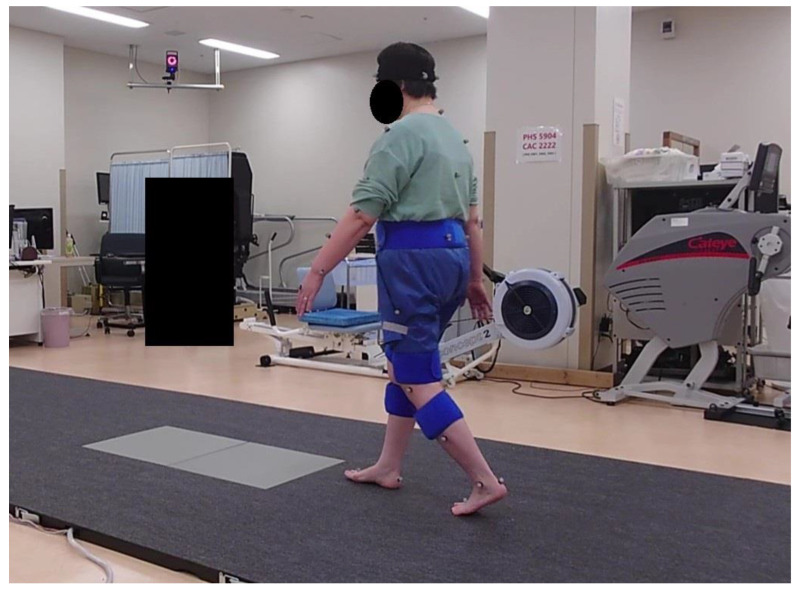
Representative gait laboratory setup. A study participant walks at self-selected speed over embedded force plates while reflective markers are tracked by a 3D optical motion capture system (100 Hz). Marker placement follows the Plug-in Gait full-body scheme; ground reaction forces are recorded synchronously and used to drive the musculoskeletal model.

Gait data were collected in a motion laboratory using a 3D optical motion capture system (Vicon Motion Systems, Oxford, UK) operating at 100 Hz with six infrared cameras synchronized with two force platforms embedded in the walkway. A representative photograph of the gait acquisition setup is shown in Figure 1. Reflective markers were placed according to the Plug-in Gait full-body scheme [21]. The participants walked at a self-selected comfortable speed and were instructed not to target the force plates. Marker trajectories and ground reactions were synchronized, visually inspected for dropouts, low-pass filtered, and time-normalized to 0–100% of the gait cycle. Two–three clean gait cycles were recorded for each participant. For inferential analysis, one valid trial per participant (free of marker loss and with complete foot strikes) was retained to avoid within-participant averaging.

### 2.5. Musculoskeletal Modeling Workflow

**Figure 2:**
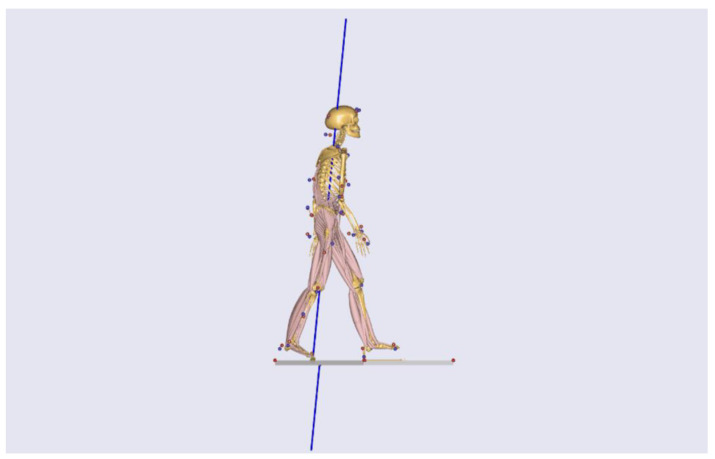
Patient-scaled AnyBody model. The AnyBody Modeling System (v6.0.5; AMMR v2.2.1) is driven by captured kinematics and ground reaction forces. Blue markers indicate experimental motion-capture markers placed on the subject. Red markers indicate the corresponding model markers in the AnyBody musculoskeletal model. Blue vectors represent ground reaction force. Intervertebral joints T12/L1–L5/S1 are modeled with three rotational degrees of freedom; inverse dynamics with static optimization (polynomial recruitment, *p* = 3) resolves muscle forces. Intervertebral disc compression force is computed along the local disc normal and normalized to body weight (%BW). The image shows a representative stance phase frame.

A three-dimensional musculoskeletal model built into the AnyBody Modeling System (AMS v6.0.5; AMMR v2.2.1) was used to estimate the internal spinal loads throughout the gait cycle. Figure 2 illustrates the patient-scaled musculoskeletal model used for AMS analyses. The base lumbar model followed the established specifications for a detailed rigid-body lumbar spine [22], and the repository resources described in the AnyBody Managed Model Repository were used [23]. The intervertebral joints from T12/L1 to L5/S1 were modeled with three rotational degrees of freedom. Patient-specific scaling was applied based on the measured body mass and height. The recorded marker kinematics drove the model through inverse kinematics, and the net joint moments were computed using inverse dynamics. The muscle forces were resolved using static optimization within the AnyBody framework [16] with a polynomial recruitment criterion (power *p* = 3, AMS default).

### 2.6. Outcome Definitions

The intervertebral disc compression force at each lumbar level (T12/L1–L5/S1) was defined as the component of the joint reaction force projected onto a local disc. The forces included contributions from external loads and modeled muscle forces and were normalized to body weight (%BW). Two summary outcomes were extracted per level: (i) peak compression force (PCF) across the gait cycle; and (ii) mean compression force over the gait cycle.

### 2.7. Grouping Strategy and Statistical Analysis

Participants were dichotomized according to passive KEA: a limitation group with knee extension limitation ≥ 10° in at least one limb, and a non-limitation group with knee extension limitation < 10°. To preserve participant-level independence and avoid attenuation of transient peak metrics by within-subject averaging, one valid trial per participant was used for inferential analyses. Distributional assumptions were assessed using the Shapiro–Wilk test. Between-group comparisons used independent-samples t-tests for approximately normal data and Wilcoxon rank-sum tests for non-normal distributions (two-sided α = 0.05). Given that multiple spinal levels were evaluated, family-wise error was controlled using the Holm procedure. Unadjusted *p*-values are reported in the text, and adjusted significance was noted where relevant. Analyses were performed using the R software (version 4.2.1; R Foundation for Statistical Computing, Vienna, Austria). Participants were grouped using a clinically meaningful cut-off of ≥10° knee-extension deficit; a sensitivity analysis using ≥5° yielded the same direction and significance at upper lumbar levels. Between-group differences are summarized with Hedges’ g (95% CI). To account for speed, we ran an ANCOVA with group as the main factor and walking speed (m/s) as a covariate.

## 3. Results

Seventeen participants were analyzed (12 women and 5 men; median age 72.0 years [range, 64.0–76.0 years]). The limitation group (*n* = 9) demonstrated larger knee extension deficits than the non-limitation group (n = 8): right KEA −12.0° vs. −5.0° (*p* < 0.001) and left KEA −12.0° vs. −5.0° (*p* = 0.015). Given *n* = 17 (9 vs. 8) at α = 0.05, the study is powered (~80%) to detect large standardized effects (Hedges’ g ≈ 1.3–1.4); smaller effects may be underpowered. Spinopelvic parameters differed, with a smaller PI (*p* = 0.006) and larger SVA (*p* = 0.041) in the limitation group. Roussouly types were distributed toward lower-PI morphologies in the limitation group (type 1–2 predominance). In the limitation group, Type 1 was observed in 3 participants and Type 2 in 4, whereas Types 3 and 4 were each represented by a single participant; in the non-limitation group, Type 1 was not observed, Types 2 and 3 were each observed in 4 participants, and Type 4 was not observed.

The overall cohort descriptors are summarized in Table 1, groupwise demographics and clinical variables in Table 2, and spinopelvic sagittal parameters in Table 3.

**Figure 3:**
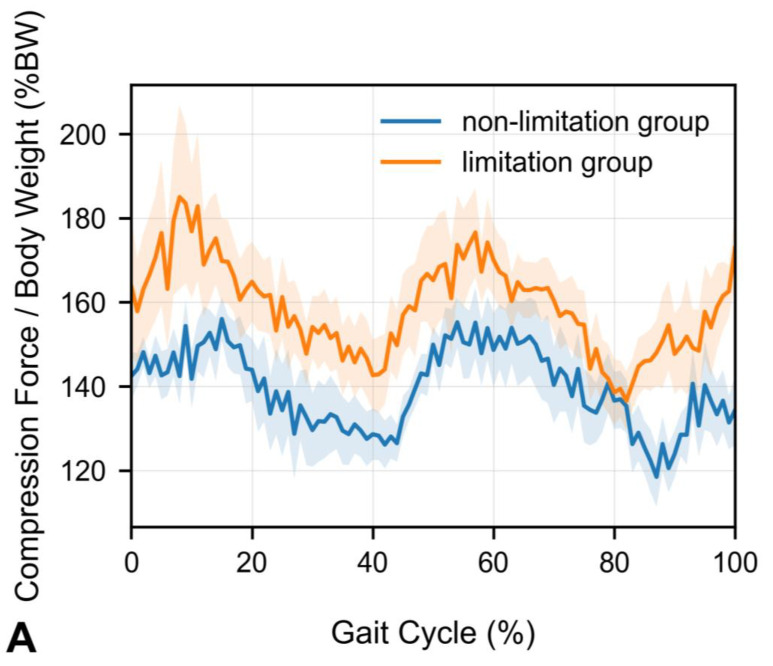

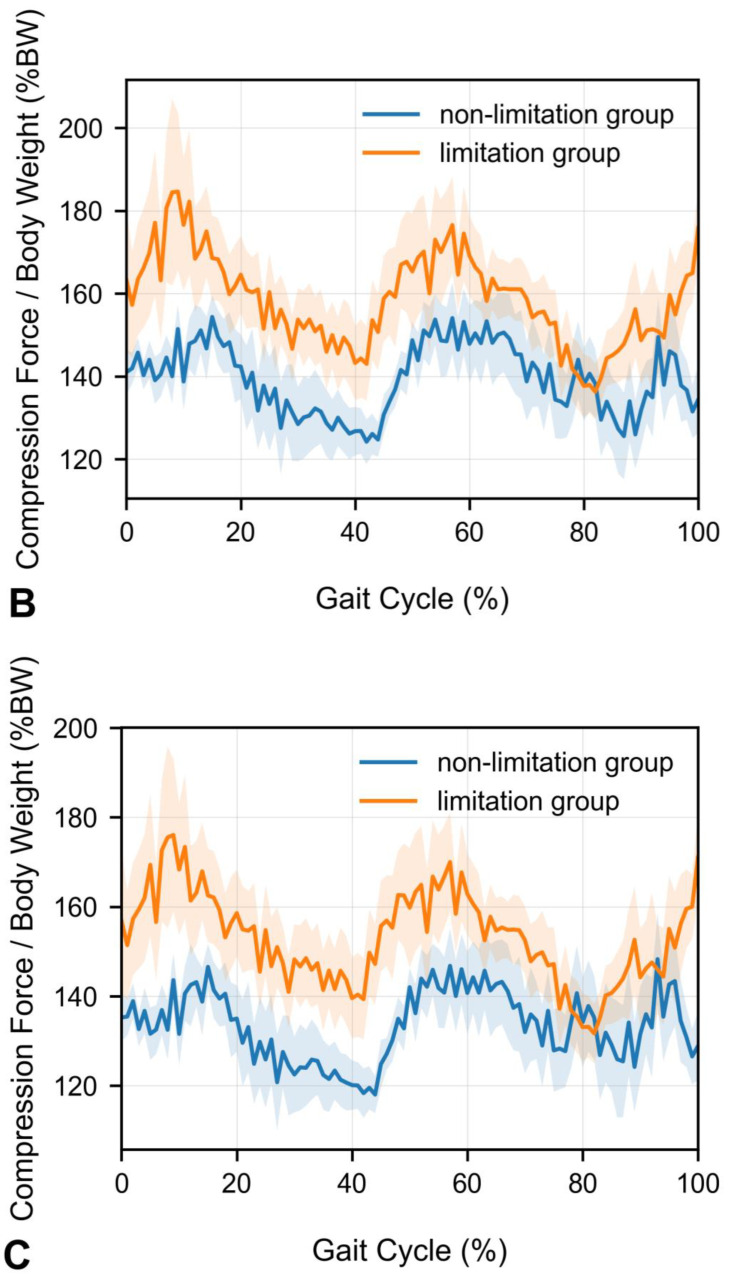

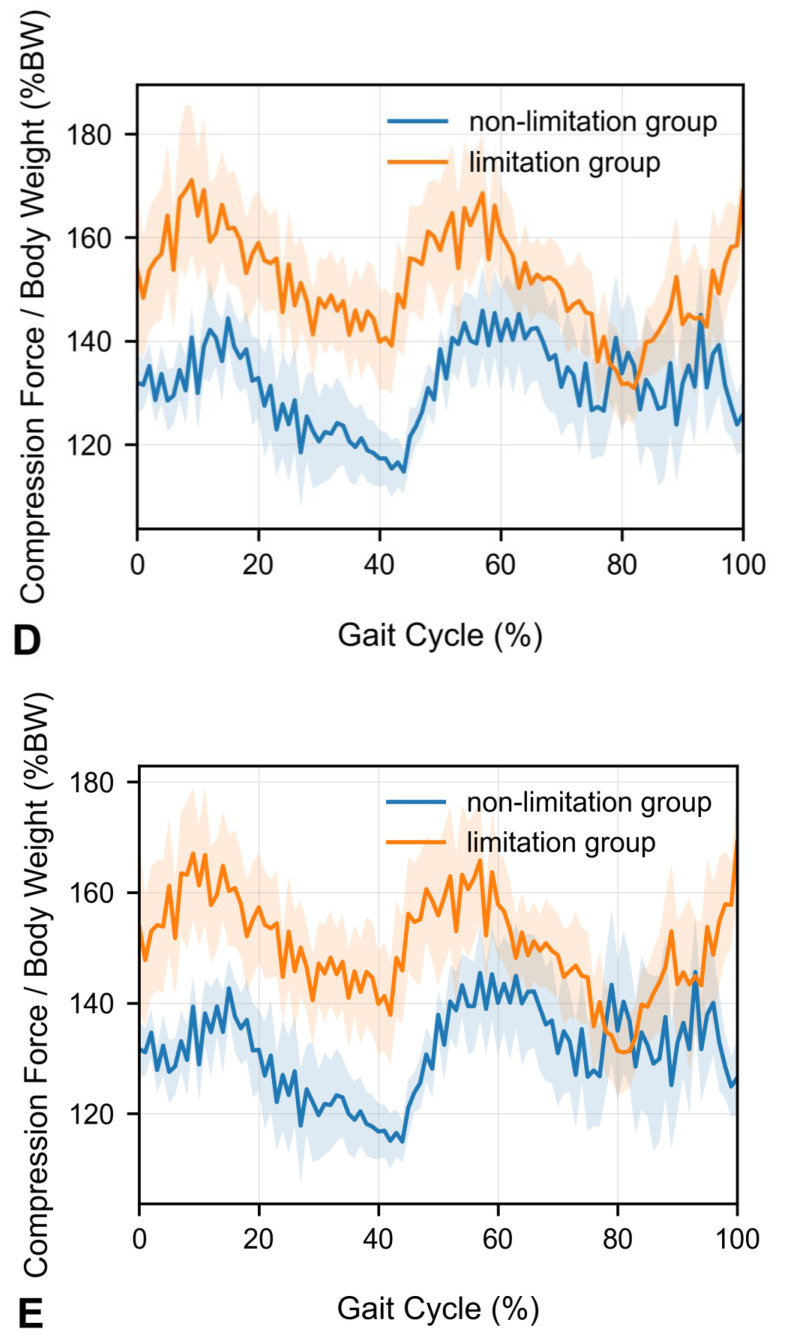

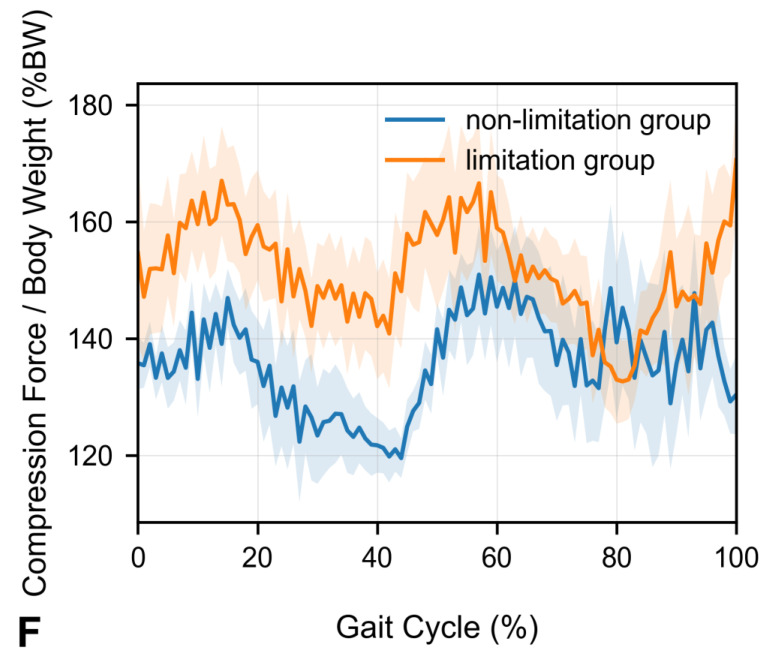
(**A**–**F**) Group-mean waveforms of lumbar disc compression force over the gait cycle (0–100%) at each level (T12/L1–L5/S1), normalized to body weight (%BW). (**A**) T12/L1, (**B**) L1/L2, (**C**) L2/L3, (**D**) L3/L4, (**E**) L4/L5, (**F**) L5/S1. Lines represent the mean across participants; shaded areas represent SEM. The non-limitation group and limitation group are overlaid for direct comparison. The *y*-axis is “Compression Force/Body Weight (%BW)” and the *x*-axis “Gait Cycle (%)”.

Group-mean waveforms of normalized lumbar disc compression (%BW) were overlaid for the limitation and non-limitation groups across the gait cycle (0–100%) at each level (T12/L1–L5/S1) (Figure 3A–F). The limitation group exhibited consistently higher trajectories across substantial portions of the gait cycle at the upper lumbar levels (T12/L1–L3/4). Waveforms are shown as mean curves with shaded standard errors of the mean (SEM).

**Figure 4:**
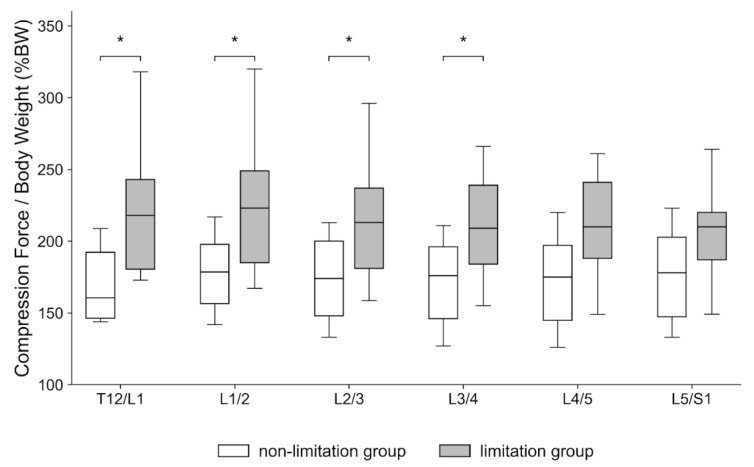
Peak compression force during the gait cycle at each spinal level (T12/L1–L5/S1), normalized to body weight (%BW). White boxes indicate the non-limitation group (knee extension angle (KEA) < 10°), and gray boxes indicate the limitation group (KEA ≥ 10°). Asterisks (*) denote *p* < 0.05 between groups (Holm-adjusted where applicable). The *y*-axis is labeled “Compression Force/Body Weight (%BW)”.

**Figure 5:**
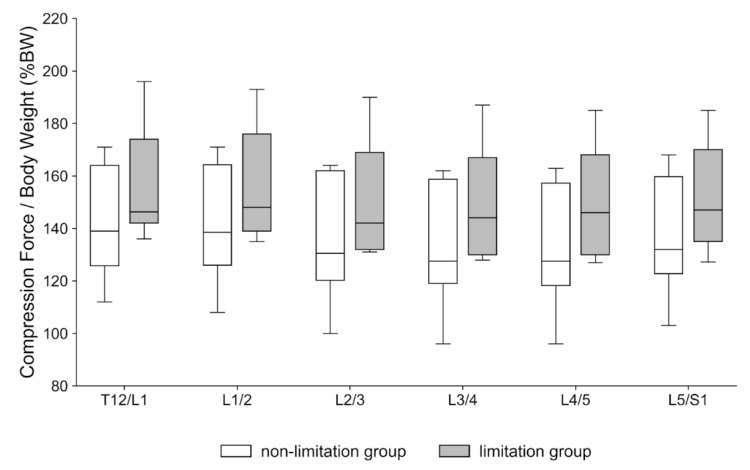
Mean compression force during the gait cycle at each level (T12/L1–L5/S1), normalized to body weight (%BW). White boxes: non-limitation group (knee extension angle (KEA) < 10°); gray boxes: limitation group (KEA ≥ 10°). No between-group differences reached statistical significance. The *y*-axis is labeled “Compression Force/Body Weight (%BW)”.

The PCF was significantly higher in the limitation group at T12/L1 (Hedges’ g = 1.314, 95% CI 0.252–2.376; *p* = 0.010, Holm-adjusted *p* **=** 0.041), L1/2 (Hedges’ g = 1.290, 95% CI 0.232–2.348; *p* = 0.011, Holm-adjusted *p* **=** 0.041), L2/3 (Hedges’ g = 1.207, 95% CI 0.161–2.252; *p* = 0.012, Holm-adjusted *p* **=** 0.041), and L3/4 (Hedges’ g = 1.060, 95% CI 0.035–2.086; *p* = 0.016, Holm-adjusted *p* **=** 0.041) (Figure 4). In contrast, the mean compression force showed no significant between-group differences across levels (Figure 5). After adjustment for walking speed, group effects remained significant at T12/L1 (ANCOVA *p* = 0.0184), L1/2 (*p* = 0.0207), and L2/3 (*p* = 0.0306), but not at L3/4 (*p* = 0.0540).

## 4. Discussion

The findings of this study demonstrate that the PCF on the upper lumbar intervertebral discs during walking is significantly increased in patients with knee extension limitations. This extends earlier observations from static postures to dynamic conditions and underscores the heightened risk of disc overload during everyday activities.

Degenerative lumbar kyphoscoliosis produces forward trunk inclination during gait, amplifying the upper lumbar loads [24]. Experimental restriction of knee extension using orthoses further confirmed this link, consistently increasing forward trunk inclination and pelvic compensation (increased posterior pelvic tilt, typically accompanied by a reduction in sacral slope) [13]. Previous static analyses have revealed that individuals with an increased sagittal vertical axis, indicating a spinal imbalance in which the body’s center of mass shifts forward, resulting in a forward-leaning or stooped posture, exhibit greater upper lumbar disc compression forces [25]. Integrating these results suggests a unifying mechanism; knee extension limitation induces compensatory forward trunk inclination, which is accentuated during walking, thereby amplifying dynamic disc loading. This distinction between the peak and average loads is critical because transient peak forces play a decisive role in disc degeneration and mechanical failure [26].

The morphological alignment patterns warrant further investigation. The limitation group exhibited a higher prevalence of Roussouly Types 1 and 2 spinal morphotypes, characterized by a low pelvic incidence, which are predisposed to degeneration and herniation at the upper lumbar and thoracolumbar levels [20,27]. The concurrence of these morphotypes with elevated dynamic loading may lead to degenerative risk.

The interplay between knee pathology and surgical intervention is clinically relevant. Outcomes following total knee arthroplasty (TKA) often depend on preoperative spinal flexibility. When flexibility is limited, abnormal knee flexion posture may persist even after technically successful surgery, sustaining abnormal spinal stress [28,29]. These insights highlight the importance of preoperative assessment that accounts for both knee and spinal alignment, as well as the potential benefits of targeted prehabilitation to optimize knee extension before TKA. Proactive addressing of knee contractures can mitigate adverse spinal loading and enhance postoperative outcomes.

From a broader perspective, knee extension limitation can be regarded as an independent risk factor of long-term lumbar degeneration. Preventive strategies should include early identification of contractures, structured rehabilitation programs, and surgical correction, where appropriate. Beyond immediate symptom relief, these interventions can play a role in slowing or preventing degenerative changes in the spine. Future longitudinal research should evaluate whether restoring knee extension leads not only to improved spinal biomechanics but also to measurable benefits in clinical outcomes, such as pain, function, and quality of life. We did not include pain, function, or imaging outcomes in this study. A prospective protocol will link gait-derived peak loads with patient-reported outcomes and imaging markers of degeneration to establish clinical relevance.

Despite offering new insights into spinal loading during walking, this study has several limitations that must be acknowledged. First, the sample size was modest, limiting its broad generalizability. External validity may be limited by the single-center design and potential healthy-volunteer bias. Nonetheless, the alignment with prior findings [13,25] reinforces confidence in the observed trends. Second, the musculoskeletal model was generic and scaled rather than patient-specific, which may not capture subtle variations in muscle activation or spinal morphology. Because the model uses anthropometric scaling by height and weight, absolute forces may be biased; our inference focuses on relative group differences, which are more robust. Future work will incorporate subject-specific curvature and trunk muscle cross-sectional areas (e.g., MRI-based) to improve biomechanical fidelity. Third, we acknowledge stride-to-stride variability in gait [30,31]. In this study we intentionally analyzed one artifact-free stride per participant and did not quantify within-subject variability or average multiple strides, which may influence estimates of peak loading. Future work will collect multi-stride data and link gait-derived peak loads with patient-reported outcomes and imaging to strengthen clinical relevance. Fourth, the study focused exclusively on biomechanical measures without a direct correlation with clinical outcomes. Longitudinal studies linking mechanical loads to degenerative progression and patient-reported outcomes are warranted. Finally, while significant differences were identified in PCF, the mean compression forces did not differ, suggesting that the pathological influence was most pronounced at peak loading points, consistent with tissue damage mechanisms [26].

## 5. Conclusions

In older adults with knee extension limitations, patient-scaled 3D musculoskeletal modeling combined with gait analysis revealed selectively higher PCF at the upper lumbar levels (T12/L1–L3/4), whereas the mean forces across the gait cycle remain unchanged. These results support knee–spine coupling, in which knee extension limitation increases forward trunk inclination and concentrates loading into short peak events during ambulation. Clinically, assessing and managing knee extension—through targeted rehabilitation and, when appropriate, perioperative planning for TKA with attention to spinal flexibility—may mitigate adverse upper lumbar loading and reduce the risk of degenerative progression. Future longitudinal and patient-specific modeling studies are warranted to determine whether restoring knee extension reduces peak spinal load and improves symptoms and function.

## Figures and Tables

**Table 1:** Overall cohort characteristics (*n* = 17).

Variable	Median	Range
Age (years)	72.0	64.0–76.0
Height (cm)	157.0	152.0–158.1
Weight (kg)	64.0	55.0–72.6
BMI (kg/m^2^)	25.5	21.9–29.9
SVA (mm)	32.3	−14.0–50.0
LL (°)	44.0	39.0–49.0
PT (°)	26.0	16.0–27.0
SS (°)	33.0	28.0–36.0
PI (°)	55.0	50.0–63.0
PI–LL (°)	14.0	6.0–21.0

Values are presented as medians and ranges. BMI, body mass index; SVA, sagittal vertical axis; LL, lumbar lordosis; PT, pelvic tilt; SS, sacral slope; PI, pelvic incidence; PI–LL, pelvic incidence minus lumbar lordosis.

**Table 2:** Participant demographics and clinical characteristics.

Variable	Limitation (*n* = 9)		Non-Limitation (*n* = 8)		*p* Value
	Median	Range	Median	Range	
Age (years)	74.0	64.0–80.0	70.5	63.3–75.0	0.766
Height (cm)	156.1	147.0–158.0	157.0	153.8–158.6	0.262
Weight (kg)	55.1	51.6–66.1	67.7	63.5–73.2	0.076
BMI (kg/m^2^)	22.5	20.5–30.2	28.4	25.1–29.6	0.191
Right KEA (°)	−12.0	−15 to −10	−5.0	−5.5 to −5.0	<0.001
Left KEA (°)	−12.0	−15 to −10	−5.0	−5.5 to −5.0	0.015

Values are presented as medians and ranges. BMI, body mass index; KEA, knee extension angle (negative = extension deficit).

**Table 3:** Spinopelvic sagittal alignment parameters.

Variable	Limitation (*n* = 9)		Non-Limitation (*n* = 8)		*p* Value
	Median	Range	Median	Range	
LL (°)	41.0	34.0–45.0	45.5	41.0–52.8	0.180
PT (°)	20.0	14.0–27.0	27.0	25.5–27.5	0.083
SS (°)	28.0	23.0–34.0	34.5	32.8–36.8	0.265
PI (°)	53.0	45.0–55.0	62.5	57.5–65.3	0.006
PI–LL (°)	14.0	3.0–15.0	12.0	7.5–22.3	0.332
SVA (mm)	50.1	32.9–56.0	17.8	9.45–26.2	0.041

Values are presented as medians and ranges. LL, lumbar lordosis; PT, pelvic tilt; SS, sacral slope; PI, pelvic incidence; SVA, sagittal vertical axis.

## Data Availability

Summary data and scripts supporting the conclusions of this article will be made available by the authors on reasonable request with institutional approval.

## Supplementary Materials

The following supporting information can be downloaded at: https://www.mdpi.com/article/10.3390/s25216605/s1, Supplementary File S1: STROBE statement.

## Abbreviations

The following abbreviations are used in this manuscript:

AMMR	AnyBody Managed Model Repository
ASD	adult spinal deformity
BW	body weight
DOF	degrees of freedom
KEA	knee extension angle
LL	lumbar lordosis
PI	pelvic incidence
PT	pelvic tilt
SS	sacral slope
SVA	sagittal vertical axis
PCF	peak compression force
